# Development and evaluation of an experimental inactivated vaccine against lumpy skin disease

**DOI:** 10.14202/vetworld.2025.3029-3040

**Published:** 2025-10-14

**Authors:** Adil Shahzad, Waseem Shahzad, Muhammad Imran Arshad, Rao Zahid Abbas, Muhammad Shahid Mahmood

**Affiliations:** 1 Institute of Microbiology, University of Agriculture, Faisalabad, Pakistan; 2 Department of Veterinary Population Medicine College of Veterinary Medicine, University of Minnesota, USA; 3 Department of Livestock and Dairy Development, Veterinary Research Institute, Punjab, Pakistan; 4 Department of Parasitology, Faculty of Veterinary Science, University of Agriculture, Faisalabad, Pakistan

**Keywords:** *Capripoxvirus*, immunogenicity, inactivated vaccine, lumpy skin disease, Montanide ISA 50 V2, rabbit model

## Abstract

**Background and Aim:**

Lumpy skin disease (LSD), caused by the LSD virus (LSDV), results in severe economic losses, reduced productivity, and restricted livestock trade. Although live attenuated vaccines are available, they pose risks such as viral shedding, recombination, and reversion to virulence. Inactivated vaccines, being safer alternatives, are particularly suitable for disease-free regions. This study aimed to develop an inactivated oil-adjuvanted vaccine using a local LSDV isolate and evaluate its immunogenicity and protective efficacy in rabbits.

**Materials and Methods:**

Scab samples were collected from clinically suspected LSD cases, and LSDV was isolated through the chorioallantoic membrane route in embryonated chicken eggs. The virus was adapted to Madin-Darby bovine kidney (MDBK) cells, inactivated with binary ethyleneimine, and formulated with Montanide Immune System Activator 50 V2 adjuvant. Sterility and safety were evaluated in laboratory animals. Twenty-four rabbits were divided into three groups: Group A received the experimental inactivated vaccine intramuscularly, Group B received a commercial live attenuated vaccine subcutaneously, and Group C served as controls. Antibody responses were assessed using enzyme-linked immunosorbent assay (ELISA) and virus neutralization tests. A challenge study with a virulent local LSDV strain was conducted to evaluate protective efficacy.

**Results:**

The inactivated vaccine elicited robust antibody responses, with ELISA sample-to-positive ratios increasing from 4.3% at baseline to 166.6% on day 42, compared with 210.1% in the live vaccine group and 6% in controls. Neutralizing antibody titers ranged from 1:32 to 1:128 (mean 1:80) in the inactivated group, compared with 1:32–1:256 (mean 1:148) in the live vaccine group, both surpassing the protective threshold (≥1:16). Post-challenge, the inactivated vaccine conferred 86% vaccine efficacy, with only mild clinical signs observed in one rabbit, while the control group developed typical LSD symptoms. No adverse reactions were recorded in vaccinated animals.

**Conclusion:**

The experimental inactivated oil-adjuvanted vaccine induced strong protective immunity in rabbits, comparable to the live attenuated vaccine but with an improved safety profile. Its inability to revert to virulence or transmit between animals makes it a promising candidate for large-scale use, especially in regions aiming to maintain disease-free status. Further evaluation in cattle under field conditions is warranted to confirm its long-term protective efficacy and potential for inclusion in control strategies.

## INTRODUCTION

Lumpy skin disease (LSD) is a highly contagious viral infection of cattle and water buffaloes caused by the LSD virus (LSDV), a member of the *Capripoxvirus* genus within the *Poxviridae* family [1]. The disease was first reported in Zambia in 1929 [2] and has since spread widely. Outbreaks have been documented across the Middle East, including Jordan, Saudi Arabia, Iraq, Lebanon, Israel, Iran, and Turkey, and in southeastern Europe, where countries such as Armenia, Greece, Russia, Azerbaijan, Albania, Serbia, Bulgaria, Kosovo, and Montenegro have reported cases since 2015 [3, 4]. By 2016, LSD had spread to several regions of southeastern Europe [5]. In Asia, the disease has emerged in Vietnam, Thailand, Bangladesh, Sri Lanka, Myanmar, India, Nepal, and Pakistan. In India, it resulted in the death of more than 155,000 cattle, while in Pakistan, the first confirmed outbreak occurred in 2022 at a cattle farm in Karachi, Sindh Province [6]. The spread is strongly linked to cross-border livestock movement, particularly from India and neighboring countries such as Malaysia and Thailand, making transboundary transmission a major concern [7].

LSD inflicts substantial economic losses globally. The incubation period ranges from 4 to 12 days, after which affected animals develop high fever (40°C–41.5°C) lasting 1–3 days, accompanied by excessive salivation, nasal discharge, lacrimation, enlarged lymph nodes, depression, reduced mobility, emaciation, and anorexia. Within 1–2 days, characteristic skin nodules appear, which become hard and necrotic. After 2–3 weeks, these nodules slough off, leaving open wounds that attract flies and predispose animals to myiasis and secondary bacterial infections. Infected bulls may shed the virus through semen for extended periods, leading to temporary or permanent infertility [8]. Due to its economic and trade implications, the World Organization for Animal Health (WOAH) classifies LSD as a notifiable disease.

In Pakistan, cattle and buffalo production is central to rural livelihoods and the national economy. According to the Economic Survey of Pakistan (2023–2024), more than 8 million people are engaged in dairy farming, deriving 35%–40% of their income from livestock. The country ranks as the world’s third-largest milk producer, with 57.5 million cattle and 46.3 million buffaloes contributing to an annual milk yield of 70 million tons (26 million tons from cattle and 42 million tons from buffaloes) [9].

LSDV transmission occurs primarily through direct contact with infected animals, although insect vectors, such as mosquitoes and flies, play a critical role in its dissemination. Outbreaks are further facilitated by livestock movement, high humidity, and rainfall. Inadequate vaccination coverage remains a major risk factor. Immunization has proven to be the most effective control strategy, supported by additional measures such as quarantine, restricted animal movement, and ring vaccination [3, 10]. Commercially available modified live vaccines are produced through blind passages in embryonated chicken eggs or cell cultures [11], and heterologous vaccination with goatpox virus or sheeppox virus (SPPV) is practiced in some regions. However, live attenuated vaccines may cause undesirable side effects, including severe swelling at the inoculation site, fever, hide damage, reduced milk yield, and occasionally mild generalized LSD (Neethling disease) [12].

To address these limitations, the European Food Safety Authority (EFSA) has recommended the use of safe, effective inactivated vaccines for controlling LSD in disease-free countries. Inactivated vaccines offer several advantages over live attenuated formulations, including improved safety, no adverse impact on milk production, and prevention of virus transmission to unvaccinated animals [13].

Despite the availability of live attenuated vaccines against LSD, their use remains controversial due to several inherent drawbacks. These include adverse post-vaccination reactions such as swelling at the inoculation site, fever, hide damage, reduced milk yield, and the occasional induction of generalized LSD (Neethling disease). Moreover, live vaccines carry the risk of viral shedding, reversion to virulence, and recombination with circulating field strains, which may compromise disease-free status in endemic and non-endemic regions alike. Although the EFSA and other international bodies strongly advocate the adoption of inactivated vaccines for safe and sustainable LSD control, very limited research has been conducted on the development and evaluation of such vaccines. Most published studies have either focused on live attenuated vaccines or heterologous vaccination using goatpox and SPPVs, with insufficient exploration of inactivated vaccine formulations for LSD. In Pakistan and neighboring countries, where LSD has emerged as a major transboundary threat to livestock productivity and trade, there is a striking lack of region-specific inactivated vaccines based on locally circulating strains. This gap is particularly concerning given that antigenic variations between field isolates and vaccine strains can significantly influence vaccine efficacy. Furthermore, while rabbits and other small laboratory animals provide a cost-effective and ethically feasible platform for preliminary immunogenicity testing, very few studies have utilized these models to evaluate the immune responses of inactivated LSD vaccines before large-scale cattle trials.

In light of these gaps, the present study was designed to isolate and molecularly characterize a local strain of LSDV from outbreak cases in Pakistan, adapt it to cell culture, and develop an inactivated oil-adjuvanted vaccine formulation. The primary aim was to evaluate the immunogenicity and protective efficacy of this experimental vaccine in rabbits by assessing antibody responses through enzyme-linked immunosorbent assay (ELISA) and virus neutralization tests (VNTs), followed by a controlled challenge study. By comparing the immune response of the inactivated vaccine with that of a commercially available live attenuated vaccine, this study sought to establish the potential of an inactivated formulation as a safer, yet immunologically effective, alternative for LSD control. Ultimately, the findings aim to provide foundational evidence for scaling up inactivated vaccine development in cattle, with the long-term goal of supporting sustainable control strategies, mitigating economic losses, and strengthening livestock health security in Pakistan and other LSD-affected regions.

## MATERIALS AND METHODS

### Ethical approval

The Director, Centre of Agricultural Biochemistry and Biotechnology/Convener, Institutional Bioethical Committee University of Agriculture, Faisalabad and the Office of Research, Innovation, and Commercialization (ORIC), University of Agriculture, Faisalabad approved this study (Approval No. 1423/ORIC, UAF). All procedures complied with the WOAH standards for animal research. The welfare of the animals, including proper housing, handling, and monitoring, was strictly ensured throughout the experiment.

### Study period and location

This study was conducted between July 2022 and June 2025 at the Institute of Microbiology, University of Agriculture Faisalabad (IOM-UAF), Veterinary Research Institute (VRI), Lahore, and the College of Veterinary Medicine, University of Minnesota (UMN), USA.

### Sample collection and processing

A total of 60 scab samples were randomly collected from suspected cases during the outbreak of LSD in Sahiwal cows (*Bos indicus*) in 20 different dairy farms located in two cattle colonies (217/Rakh Branch canal [RB] and 225/RB) in Faisalabad, Pakistan. Scabs were collected from cows with high fever, excessive salivation, bilateral epiphora, skin edema, and enlarged lymph nodules. The area was cleaned before sample collection, and the hair was clipped. This was an exploratory immunogenicity study following standard sample sizes used in similar animal trials. The scabs were labeled and placed in a sterile jar containing a medium for virus transport. All samples were transported to the Cell Culture Laboratory, IOM, Faisalabad University of Agriculture, Pakistan. All sample processing and experimental procedures were conducted under Biosafety Level 2 containment conditions, following established biosafety protocols and institutional guidelines.

### Isolation of viruses in embryonated chicken eggs

The scabs were cut into small pieces using sterile scissors and blades. Using a pestle and mortar, the scabs were homogenized in 10 mL of sterile phosphate buffer saline (PBS) containing streptomycin sulfate (1 mg/mL), benzyl penicillin (1,000 IU/mL), and amphotericin B (100 μL/mL). The suspension was centrifuged at 4,200 × *g* for 30 min at 4°C, and the supernatant was collected into sterile vials, followed by filtration through a 0.45 mM filter. The filtrate was inoculated into embryonated chicken eggs through the chorioallantoic membrane (CAM) route [14]. After incubation at 37°C, the CAM from all eggs was checked for pock lesions (dark opaque necropsied large areas about 3–5 mm in diameter) and compared with the CAM from the negative control eggs. The CAM was homogenized in PBS, followed by centrifugation at 4,200 × *g* for 30 min at 4°C. Aliquots of supernatant containing the virus isolated from field samples were stored at −80°C until further use. A local LSDV isolate was used to ensure that the vaccine antigen closely matches circulating field strains, thereby enhancing strain-specific immunity and minimizing the risk of antigenic mismatch.

### Molecular identification of the LSDV

DNA was extracted from the CAM supernatant using the Gene Jet Genomic DNA Purification Kit (Thermo Scientific, Lithuania) at the Research and Development Division, VRI, Lahore, Punjab, Pakistan. The primers (Table 1) [15–17] used to amplify targeted gene segments of LSDV P32 [15, 16], and LSDV G-protein coupled receptors (*GPCR*) genes [17]; P32 is an envelope protein gene, while the *GPCR* gene plays a significant part in host interaction and facilitating immune invasion. This was followed by polymerase chain reaction (PCR) to confirm the presence of the virus. I-CYCLER BIORAD DSR-3/175 was used to amplify the specific *LSDV* gene regions. Thermal conditions for *P32* gene amplification included an initial denaturation at 95°C for 5 min, followed by denaturation at 95°C for 30 s. Annealing was then performed at 58°C for 30 s, and the extension step was performed at 72°C for 45 s. The final elongation occurred at 72°C for 5 min. PCR thermal conditions for amplification of the *GPCR* gene were initial denaturation at 95°C for 15 min, followed by denaturation at 94°C for 1 min. Annealing was performed at 48°C for 1 min, and the extension step was performed at 72°C for 1 min. The final elongation was performed at 72°C for 10 min. The PCR products were separated based on their size and charge using a 1.2% agarose gel and a DNA ladder of 100 bp. An electric current was provided to the DNA loaded in the gel. Negatively charged DNA fragments traveled through the gel toward the positive side of the gel electrophoresis apparatus.

**Table 1:** Primers used to amplify the specific *LSDV* gene regions.

Target gene	Primers sequence (5′-3′)	Amplification size (bp)	References
P32-F	TTTCCTGATTTTTCTTACTAT	192	[15, 16]
P32-R	AAATTATATACGTAAATAAC		
GPCR-F	TGAAAAATTAATCCATTCTTCTAAACA	684	[17]
GPCR-R	TCATGTATTTTATAACGATAATGCAAA		

LSDV = Lumpy skin disease virus, GPCR = G-protein coupled receptors, F = Forward, R = Reverse

### Sequencing and GenBank submission

The gene sequence data for the *P32* gene were permanently archived in the National Center for Biotechnology Information (NCBI) GenBank database under accession number PQ720422. The *GPCR* gene was sequenced at the College of Veterinary Medicine, UMN, Saint Paul, Minnesota, USA. The resulting sequence data have been submitted and archived in the NCBI GenBank under accession numbers PV288761–PV288762.

### Adaptation of LSDV isolates to cell cultures

Madin-Darby bovine kidney (MDBK) cells were used to propagate and titrate the LSDV cells. The key steps include serial passaging of the local virus isolate on MDBK cells, monitoring cytopathic effects (CPEs), and confirming robust replication. Cell adaptation is critical to achieve high-yield production under controlled, scalable, and serum-free conditions. The cells were grown at 37°C with 5% CO_2_ in Glasgow Minimum Essential Medium (GMEM) enriched with 5% (v/v) tryptose phosphate broth (TPB) and 10% (v/v) fetal bovine serum (FBS). The cultures were maintained under these conditions until they reached approximately 90% confluency, at which point they were used for LSDV propagation. Cell monolayers were infected with LSDV and incubated at 37°C with 5% CO_2_. The monolayers were examined daily for the appearance of CPE. The CPE appeared on the 5^th^ day post-infection and peaked at 9–10 days. Three blind passages were performed, and the 4^th^ passage material was used for vaccine production.

### Virus titration [50% tissue culture infective dose; TCID_50_] calculation

The confirmed LSDV was typically isolated and quantified (TCID_50_) using primary culture. LSDV infects MDBK cells and forms multifocal areas of hyperplastic cells [8]. The confirmed LSDV was adapted in MDBK cell lines for vaccination preparation. The viral titer was calculated using the median tissue culture infectious dose assay [18]. For virus titration, LSDV was adapted in MDBK cells at a multiplicity of infection of 0.01 and passaged thrice to ensure optimal replication. Tenfold serial dilutions were prepared by transferring 100 μL of virus into 900 μL of GMEM across eight tubes. MDBK cells were seeded in 96-well plates and incubated at 37°C with 5% CO_2_ until monolayers formed. The diluted virus samples were inoculated into the respective wells (eight replicates per dilution) and incubated for 7 days. The cytotoxic effects (CPE) were recorded daily, and the TCID_50_ was calculated using the Reed-Muench method, which estimates the viral dilution at which 50% of the wells exhibit CPE, indicating infectivity.

### Virus inactivation and preparation of vaccines

The virus at passage level 4 was inactivated using 0.03/mL of binary ethyleneimine (BEI) [13, 19]. BEI, an aziridine, alkylates viral nucleic acids while preserving protein conformation and antigenic epitopes, offering superior antigenicity compared to formaldehyde. Complete virus inactivation was confirmed through three consecutive passages in MDBK cell cultures. Following inactivation and incubation for 24 h at 37°C, sodium thiosulfate was added at 10 mL/100 mL. This was followed by reincubation for 24 h at 37°C. Montanide Immune System Activator (ISA)-50 V2 adjuvant (SEPPIC, France) @ 50%, thiomersal 0.003%, and penicillin 4,000,000 IU @ 0.1 mL/100 mL were added to this inactivated virus. Montanide ISA-50 V2 was selected for its ability to form a stable water-in-oil emulsion, enhance antigen retention, and stimulate a robust, long-lasting immune response. Compared with aluminum hydroxide, which mainly induces humoral immunity, ISA-50 V2 promotes both humoral and cellular immune responses, which are critical for effective protection against LSDV. Its established safety and efficacy in veterinary vaccines make it a preferred adjuvant for inactivated formulations [20].

### Vaccine sterility and safety testing

The sterility of the vaccine was verified by inoculating it in thioglycolate broth and then incubating it at 35°C for 14 days. Sabouraud dextrose agar was used to check for fungal growth. Nutrient agar, nutrient broth, and MacConkey’s agar were also used to check for bacterial growth [21]. A thorough safety evaluation of the experimental inactivated oil-adjuvanted LSD vaccine was performed along with sterility testing. This evaluation involved administering the vaccine to a cohort of laboratory animals, including five mice, five guinea pigs, and two young male calves. The animals were then closely observed for a duration of 4 weeks, during which comprehensive clinical monitoring was conducted. The monitoring focused on detecting any signs of adverse reactions, changes in behavior, or overall health status, thereby ensuring a detailed assessment of the vaccine’s safety and tolerability. This careful and systematic approach was crucial to establishing the safety profile of the vaccine before advancing to subsequent stages of testing.

### Experimental animals: Grouping, housing, and management

Rabbits were used as a preliminary immunogenicity model due to their better ethical feasibility and lower cost. To evaluate the protective efficacy of the inactivated oil-adjuvanted LSD vaccine, 24 rabbits of mixed gender (12–14 weeks of age, weighing 2.7–3.0 kg) were randomly allocated to three different groups (Groups A, B, and C; eight rabbits per group). On arrival, the rabbits underwent a 7-day acclimation period, during which their general health was monitored daily before the trial started. All rabbits were housed in a clean, well-ventilated animal facility under standardized conditions. Animals were kept in individual cages to prevent cross-contamination between groups. The temperature and humidity were maintained within the recommended limits. Standard pellet feed and clean drinking water were provided *ad libitum*. Regular health monitoring was conducted throughout the study to ensure the welfare of the animals and to observe any clinical signs that may occur post-vaccination and following challenge.

### Vaccination protocol

Group A rabbits were inoculated intramuscularly with 0.2 mL of oil-adjuvanted, inactivated vaccine prepared in this study. Group B rabbits were vaccinated subcutaneously with 0.2 mL of commercially available live attenuated vaccine. Group C served as the negative control group. All animals were monitored daily for 21 days for any clinical signs. The vaccine booster dose was administered on day 22, and the trial ended on day 42. No significant local or systemic reactions were observed in the post-vaccination groups of rabbits.

### Serological testing (ELISA)

An indirect ELISA test was performed to detect antibodies in sera against LSDV induced by vaccination against LSD in rabbits [13, 18]. Serum samples were analyzed in triplicate for LSDV antibodies using the ID Screen^®^ Capripox Double Antigen ELISA (ID.vet, Grabels, France, a validated, OIE-recommended serological test kit for detecting *Capripoxvirus* antibodies in cattle, sheep, goats, and other susceptible species) following the manufacturer’s instructions. The optical density (OD) was measured at 450 nm. The sample-to-positive (S/P) ratio was calculated as follows:

S/P% = [(OD sample – OD negative)/(OD positive – OD negative)] × 100

Samples with S/P% <30% were classified as negative, while ≥30% were considered positive [22]. The differences in antibody titers obtained after vaccination were statistically analyzed using a complete randomized design, and the means of antibody titers were compared using a *post hoc* technique, specifically Dunnett’s test.

### VNT

A VNT was performed using serum samples collected from rabbits on day 42 post-immunization to evaluate the ability of the antibodies induced by vaccination to neutralize the LSDV. Blood samples from all experimental groups, namely, Group A (inactivated vaccine), Group B (live attenuated commercially available vaccine), and Group C (unvaccinated controls), were processed to obtain serum, which was then heat-inactivated at 56°C for 30 min to eliminate complement activity. The neutralizing antibody titers were determined using the Reed-Muench method, recorded as the highest serum dilution at which 50% of the cell culture wells exhibited complete inhibition of CPE. Each serum sample was subjected to serial two-fold dilutions, ranging from 1:2 to 1:1024. Equal volumes of diluted serum and LSDV containing approximately 100 TCID_50_ were mixed and incubated at 37°C for 1 h to allow virus-neutralizing antibody interaction. These virus-serum mixtures were then inoculated onto MDBK cell monolayers prepared in 96-well tissue culture plates. Plates were maintained at 37°C in a humidified atmosphere with 5% CO_2_ and monitored daily for up to 7 days for CPE.

### Challenge study in rabbits

VE was determined by the formula, VE = 1 – Relative Risk. Rabbits in all three groups were observed for 42 days post-vaccination to monitor immune response and ensure the absence of vaccine-induced adverse effects. After this period, the animals were challenged with a virulent local LSDV field strain. The virus was administered intradermally on the inner thigh, using 0.2 mL of inoculum containing 10^5^ TCID_50_ per rabbit, under aseptic conditions. Animals were observed daily for 14 days post-challenge for clinical signs including fever, local swelling, skin nodules, and behavioral changes.

## RESULTS

### Molecular characterization of LSDV and GenBank submissions

**Figure 1:**
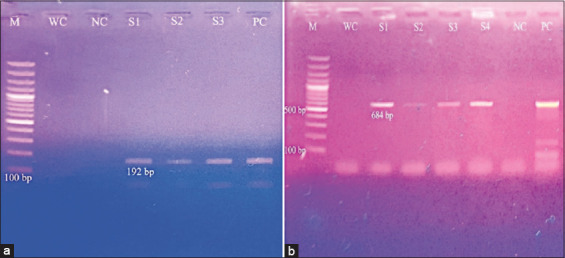
PCR confirmation of lumpy skin disease virus using gene-specific primers for (a) P32 and (b) GPCR = M, 100 bp marker, WC = Water control, NC = Negative control, PC = Positive control, S1–S4 = Field samples. PCR = Polymerase chain reaction, GPCR = G-protein coupled receptors.

The LSDV isolated in this study was confirmed by conventional PCR using *P32* and *GPCR* genes (Figure 1). The gene sequence data for the *P32* gene were permanently archived in the NCBI GenBank database under accession number PQ720422.

Gene sequencing analysis was performed at the College of Veterinary Medicine, UMN, Saint Paul, USA, to confirm the *GPCR* gene. The sequence data were permanently archived in the NCBI GenBank database under accession numbers PV288761–PV288762 and BankIt ID: 2901800.

### Virus propagation and titration (TCID_50_ calculation)

**Figure 2:**
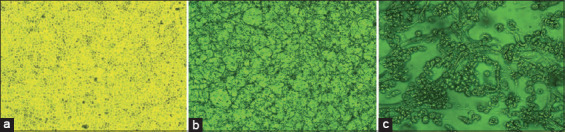
Cytopathic effects (CPEs) of lumpy skin disease virus on Madin-Darby bovine kidney cells: (a) Uninfected control, (b) infected cells showing CPE at 100×, and (c) infected cells showing CPE at ×400.

Molecularly confirmed LSDV was adapted to MDBK cells over four passages. Cells were cultured in GMEM containing 5% TPB and 10% FBS at 37°C under 5% CO_2_. At ~90% confluency, monolayers were infected with LSDV for propagation and titer assessment (Figure 2). At each passage, the virus was confirmed by PCR using the *P32* and *GPCR* genes. CPE in the wells was observed and recorded, as presented in Table 2.

**Table 2:** Visualization of the CPE in the wells.

Log_10_ dilution	Ni (number of wells used)	Ri (well positive with CPE)	P (positive proportion) = ri/ni	1-P
10^−1^	10	10	01	0
10^−2^	10	10	01	0
10^−3^	10	10	01	0
10^−4^	10	10	01	0
10^−5^	10	10	01	0
10^−6^	10	05	0.5	0.5
10^−7^	10	00	0.0	01
10^−8^	10	00	0.0	01

CPE = Cytopathic effect

The virus titer was recorded as 10^7.0^ TCID_50_/mL. Titer was calculated by the following formula:

TCID_50_ = (x_o_-d/2) + d (∑ri/ni 0f (1-P)

Where X_0_ = wells with 100% CPE and d = dilution factor.

### Vaccine preparation: Virus inactivation and adjuvant addition

The virus was inactivated using BEI [13]. Virus inactivation was verified by the lack of CPEs after three repeated passages in MDBK cells, followed by the addition of Montanide ISA-50 V2 adjuvant (SEPPIC) to prepare the oil-adjuvanted inactivated vaccine against LSD.

### Vaccine sterility and safety evaluation

The sterility test was performed using the direct inoculation technique. Thioglycolate medium was used to check aerobic and anaerobic bacterial growth after incubation at 35°C for 14 days. Sabouraud dextrose agar was used to check fungal growth. Nutrient agar and nutrient broth were used to assess bacterial growth, while MacConkey agar was used to examine the growth of Gram-negative bacteria [21]. The vaccine was found to be free of bacterial and fungal contamination.

In addition to sterility testing, the safety of the experimental inactivated oil-adjuvanted LSD vaccine was evaluated. This involved the administration of the vaccine to five laboratory mice, five guinea pigs, and two young male calves (young stock), followed by careful clinical monitoring for 4 weeks. No signs of local or systemic adverse reactions, mortality, or clinical abnormalities were recorded in any of the test animals during the observation period. Collectively, these results confirmed that the vaccine was not only sterile but also safe for use in target and non-target species under the conditions tested.

### Evaluation of antibody titers and immune response

The ELISA results were interpreted using the formula:

(OD sample in each well − OD negative control)/(OD positive control −OD negative control) = Answer × 100 = S/P ratio

Here, S denotes the difference between the sample and negative control ODs, and P denotes the difference between the positive and negative control ODs. A ratio of <30% indicates a negative result, whereas ≥30% indicates a positive result.

Antibody titers were assessed in three groups: Group A (experimental inactivated oil-adjuvanted LSD vaccine), Group B (commercially available vaccine), and Group C (control group) at days 0, 22, and 42.

- Group A: S/P% values were 4.3 (day 0), 71.5 (day 22), and 166.6 (day 42), with a total mean titer of 1333
- Group B: S/P% values were 5.9 (day 0), 93.8 (day 22), and 210.1 (day 42), with a total mean titer of 1681
- Group C: S/P% values were 5.8 (day 0), 6.3 (day 22), and 6.0 (day 42), with a total mean titer of 48.

**Figure 3:**
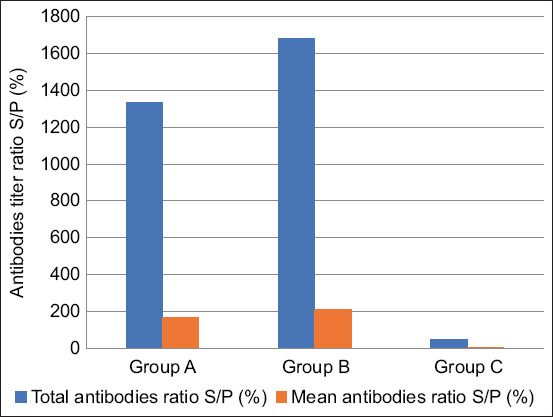
Total and mean antibody titers (sample-to-positive ratio) at 42 days after vaccination.

These results (Table 3 and Figure 3) indicated that both vaccines induced strong antibody responses, while the control group remained negative.

**Table 3:** Antibody titers measured by indirect enzyme-linked immunosorbent assay.

S. No. (Rabbits)	Group A experimental inactivated oil adjuvant LSD vaccine 0.2 mL (I/M) S/P ratio (%)			Group B commercially available vaccine 0.2 mL (S/C) ratio S/P (%)			Group C (Control group) Ratio S/P (%)		
		
	Zero day	22 Days	42 Days	Zero Day	22 Days	42 Days	Zero day	22 Days	42 Days
1	4	108	234	5	129	280	4	4	7
2	3	98	188	4	108	196	8	6	5
3	4	20	23	9	23	169	5	7	9
4	5	109	254	7	111	271	7	1	2
5	3	102	267	3	13	21	4	6	3
6	5	14	19	5	180	297	8	9	8
7	3	98	204	8	99	253	3	9	9
8	7	23	144	6	87	194	7	8	5
Total S/P ratio (%)	34	572	1333	47	750	1681	46	50	48
Average antibody titers of each group	4.3	71.5	166.6	5.9	93.8	210.1	5.8	6.3	6
Percentage of positive samples	0	62.5	75	0	75	87.5	0	0	0

S/P = Sample-to-positive, LSD = Lumpy skin disease

### Virus neutralization titers

The VNT was conducted 42 days post-vaccination to evaluate the presence and strength of LSDV-specific neutralizing antibodies in serum samples (Table 4).

**Table 4:** Virus neutralization titers.

S. No.	Group A (inactivated vaccine)	Group B: commercially available live attenuated vaccine	Group C (unvaccinated group)
1	1:128	1:256	<1:2
2	1:64	1:128	<1:2
3	1:32	1:64	<1:2
4	1:128	1:256	<1:2
5	1:64	1:32	<1:2
6	1:32	1:256	<1:2
7	1:64	1:64	<1:2
8	1:128	1:128	<1:2
Mean	1:80	1:148	<1:2

- Group A (Inactivated vaccine, Intramuscular): Titers ranged from 1:32 to 1:128, with a mean neutralization titer of 1:80. All animals exceeded the protective threshold (≥1:16)
- Group B (Commercial live vaccine, Subcutaneous): Titers ranged from 1:32 to 1:256, with a mean neutralization titer of 1:148, indicating stronger immunogenicity
- Group C (Control): All titers <1:2, showing no protective response.

These results confirm that both vaccinated groups developed virus-specific neutralizing antibodies, with Group B eliciting a stronger response, but Group A achieving protective immunity.

### Challenge study in rabbits

All rabbits were monitored for 42 days post-vaccination to assess immune response and confirm the absence of adverse effects. They were then intradermally challenged with 0.2 mL of virulent LSDV (10^5^ TCID_50_) and observed for 14 days. Only one rabbit in the inactivated vaccine group showed mild swelling, while all animals in the live vaccine group remained clinically normal. In contrast, seven of eight rabbits in the unvaccinated group developed typical LSD symptoms, confirming the virulence of the challenge strain.

Vaccine efficacy (VE) was calculated using the formula:

VE = 1 – Relative risk

The developed inactivated vaccine demonstrated a VE of 86%.

### Statistical analysis

Statistical analysis was performed using Dunnett’s *post hoc* test to compare mean antibody titers. The D-value calculation revealed significant differences between groups. The recorded differences between means were 1285 and 1633 in Groups A and B, respectively, both greater than the D-value (77.78), confirming significant immune responses compared with controls.

The standard deviations for Group A (40.30%) and Group B (47.55%) reflected moderate variability, while Group C had 0% variability. Variance within Groups A and B was 0.70 and 1.13, respectively, with a pooled variance of 0.91 and a standard error of 0.48.

At 42 days post-vaccination, virus neutralization results confirmed protective responses in Groups A (mean titer 1:80; log_2_ = 6.13) and B (mean titer 1:148; log_2_ = 6.88), while Group C had no detectable titers (<1:2). Dunnett’s test yielded t-values of 12.84 (Group A) and 14.41 (Group B), both highly significant (p < 0.001).

These results confirm that both vaccines induced statistically significant and protective neutralizing antibody responses, with the live vaccine generating a stronger immunogenic effect.

## DISCUSSION

### Global emergence and threat of LSD

Recent LSD outbreaks in Europe, the Middle East, and central Asia [13] pose a serious threat to livestock farming, particularly cattle farming in Pakistan. Pakistan ranks as the third-largest milk producer globally, and the cattle and buffalo farming sector is crucial for both livelihoods and the national economy. In fiscal year 2024, livestock contributed 60.84% to the agricultural sector and 14.63% to the national GDP.

While LSD was once confined primarily to Africa, its spread to neighboring countries such as India, China, and Iran poses a direct risk to Pakistan’s livestock sector. With the second-largest livestock population globally and an economy heavily reliant on agriculture, Pakistan faces severe implications if LSD outbreaks occur. The potential for LSD to cross borders from neighboring countries further heightens the risk to its livestock industry.

### Risk factors and disease entry into Pakistan

The primary risks for LSD entering Pakistan include uncontrolled livestock movement across borders and insufficient vector control measures. Effective management strategies such as quarantine protocols, vector control, and vaccination are essential to mitigate these risks. According to Khan *et al*. [23], LSD was not confirmed in Pakistan until 2021, with the first outbreak reported in 2022 at a cattle farm in Karachi, Sindh [6].

Pakistan’s geographic position makes it vulnerable to cross-border incursions of the virus. Although government institutions are prepared to handle infectious diseases and vaccination programs, the financial burden of large-scale vaccination presents a challenge. While the Livestock Department covers vaccine costs, the economic situation limits the extent of such interventions. Complete eradication may be difficult given Pakistan’s border dynamics and large livestock population. Therefore, controlling spread through vector management, regulating animal movements, and vaccinating livestock remains crucial. Veterinary vaccines are essential not only for animal health and livestock productivity but also for food safety and public health [24].

### Limitations of current live vaccines and the role of inactivated vaccines

Farmers are often reluctant to use live attenuated vaccines due to concerns about side effects. In contrast, inactivated vaccines are valued for their safety, stability in tropical environments, and ability to be incorporated into polyvalent formulations. They can be used in disease-free regions without compromising status and do not pose risks of reversion to virulence or transmission between animals. These advantages make inactivated vaccines particularly significant for high-risk countries such as Pakistan, where cattle farming plays a central role in disease eradication efforts [13].

Until now, no inactivated vaccine for LSD has been developed or tested. However, a recent study by Boumart *et al*. [25] successfully tested an inactivated sheep pox vaccine, another *Capripoxvirus*, and demonstrated complete protection against a virulent strain, with robust and long-lasting antibody responses.

### Molecular and serological diagnostics for LSD

Several molecular techniques, including conventional PCR, real-time PCR, and high-resolution melting assays, are employed to detect LSDV. These target genomic regions, such as GPCR, RPO30, P32, and extracellular enveloped virus (EEV), provide insights into viral evolution and dissemination [26].

Accurate and timely diagnostics are essential for LSD control. Serological methods such as the VNT, indirect fluorescent antibody test, and ELISA are widely used. Although reliable, these tests sometimes fail to distinguish between parapoxvirus and *Capripoxvirus*. However, the ID Screen^®^ Capripox Double Antigen ELISA can detect *Capripoxvirus* antibodies without cross-reactivity [22].

This ELISA kit (ID.vet) was used in the present field trial. Serum samples were incubated on *Capripoxvirus* antigen-coated plates, processed with conjugate and substrate solutions, and analyzed at 450 nm. Samples were classified as negative when the S/P ratio was <30% and positive when ≥30% [13]. In addition, the rP32-based indirect ELISA effectively detected antibodies in vaccinated animals. Given Pakistan’s vulnerability and the economic burden of LSD, reliable serological assays are essential [27]. Several viral infections have already severely affected female buffaloes and cattle in Pakistan [28], making vaccination a critical intervention [29].

### Key findings

Both experimental and commercial vaccines induced strong immune responses, with statistically significant differences compared with the control group. Variability in immune responses was observed between vaccinated groups, with the commercial vaccine eliciting more diverse responses than the experimental vaccine. The control group exhibited no immune response, confirming vaccination as the source of protective immunity.

The inactivated vaccine group achieved a mean neutralization titer of 1:80 (log_2_ mean = 6.13), indicating consistent protection across all animals. The live attenuated vaccine group produced a higher mean neutralization titer of 1:148 (log_2_ mean = 6.88), reflecting greater potency. The control group showed no detectable neutralizing antibodies (<1:2), confirming susceptibility.

Statistical analysis revealed highly significant differences (p < 0.001) between vaccinated groups and controls, highlighting the strong efficacy of both formulations. Although the live vaccine induced a more robust response, the inactivated vaccine elicited protective antibody levels above the threshold, while avoiding the risks associated with live vaccines.

## CONCLUSION

This study successfully developed and evaluated an experimental inactivated oil-adjuvanted vaccine against LSDV using a local isolate. The vaccine elicited a strong immune response in rabbits, with antibody titers (S/P%: 4.3→166.6) and mean neutralization titers (1:80) significantly higher than the control group and above the protective threshold. The commercial live attenuated vaccine produced a stronger neutralizing response (mean titer 1:148), but both vaccine formulations provided effective protection. Post-challenge, the inactivated vaccine demonstrated 86% vaccine efficacy, with only one animal showing mild signs, while all controls developed typical LSD symptoms.

The results highlight the potential of an inactivated vaccine as a safer alternative to live attenuated vaccines, particularly in regions where live vaccines raise concerns of viral shedding, reversion to virulence, or interference with trade. The absence of adverse effects, combined with stable immunogenicity, underscores the suitability of inactivated vaccines for large-scale use in disease-endemic and disease-free regions alike. Adoption of this vaccine could strengthen livestock health security, reduce economic losses, and enhance food security in countries such as Pakistan, where livestock contributes substantially to national GDP and livelihoods.

This study demonstrated several strengths, including being the first to develop an inactivated LSDV vaccine based on a locally isolated strain in Pakistan, showing consistent protective immunity in rabbits under controlled conditions, and employing Montanide ISA-50 V2 adjuvant to enhance both humoral and cellular immune responses. Furthermore, it provided an ethical and cost-effective evaluation in a small animal model before large-scale trials in cattle. However, certain limitations should be acknowledged. The study was conducted in rabbits, rather than the natural host, which may not fully replicate the protection observed in cattle. The duration of immunity was not assessed, and long-term protection remains unverified. In addition, the controlled experimental conditions did not account for field-level factors, such as vector pressure or herd management, and cost-effectiveness comparisons between live and inactivated vaccines were not evaluated.

Future research should focus on validating safety, efficacy, and duration of immunity through cattle field trials. Studies should also investigate cross-protection against antigenic variants, potential polyvalent formulations, and the integration of this vaccine into national control programs. Long-term assessments of booster requirements, herd immunity dynamics, and economic feasibility are also critical for adoption. The development of differentiating between infected and vaccinated animals using compatible inactivated vaccines could further aid surveillance and international trade.

This study provides strong preliminary evidence that an inactivated oil-adjuvanted vaccine derived from a local LSDV isolate can induce protective immunity comparable to live attenuated vaccines while offering a superior safety profile. Although further validation in cattle is required, these findings underscore the potential of inactivated vaccines as a pivotal tool in LSD control strategies. Adoption of such vaccines could safeguard Pakistan’s livestock sector, protect farmer livelihoods, and contribute to regional efforts in mitigating the transboundary spread of LSD.

## DATA AVAILABILITY

The supplementary data can be available from the corresponding author upon request, subject to rational justification.

## AUTHORS’ CONTRIBUTIONS

AS and MSM: Conceptualization, methodological design, and optimization. AS, WS, MSM, and MIA: Data collection, analysis, and interpretation. AS: Drafted the manuscript. AS, MSM, and RZA: Reviewed and edited the manuscript. WS, RZA, MIA, and MSM: Administration and study supervision. All authors have reviewed and approved the final version of the manuscript.
